# Trehalose for Ocular Surface Health

**DOI:** 10.3390/biom10050809

**Published:** 2020-05-25

**Authors:** Jarmo Laihia, Kai Kaarniranta

**Affiliations:** 1 Finnsusp Ltd., Pääskykalliontie 5, FI-21420 Lieto, Finland; 2 Department of Ophthalmology, Institute of Clinical Medicine, University of Eastern Finland, FI-70210 Kuopio, Finland; kai.kaarniranta@uef.fi; 3 Department of Ophthalmology, Kuopio University Hospital, FI-70210 Kuopio, Finland

**Keywords:** trehalose, dry eye syndromes, oxidative stress, autophagy, inflammation, cytoprotection, randomized controlled trials, animal models, molecular chaperones, patents

## Abstract

Trehalose is a natural disaccharide synthesized in various life forms, but not found in vertebrates. An increasing body of evidence demonstrates exceptional bioprotective characteristics of trehalose. This review discusses the scientific findings on potential functions of trehalose in oxidative stress, protein clearance, and inflammation, with an emphasis on animal models and clinical trials in ophthalmology. The main objective is to help understand the beneficial effects of trehalose in clinical trials and practice, especially in patients suffering from ocular surface disease. The discussion is supplemented with an overview of patents for the use of trehalose in dry eye and with prospects for the 2020s.

## 1. Introduction

**Figure 1:**
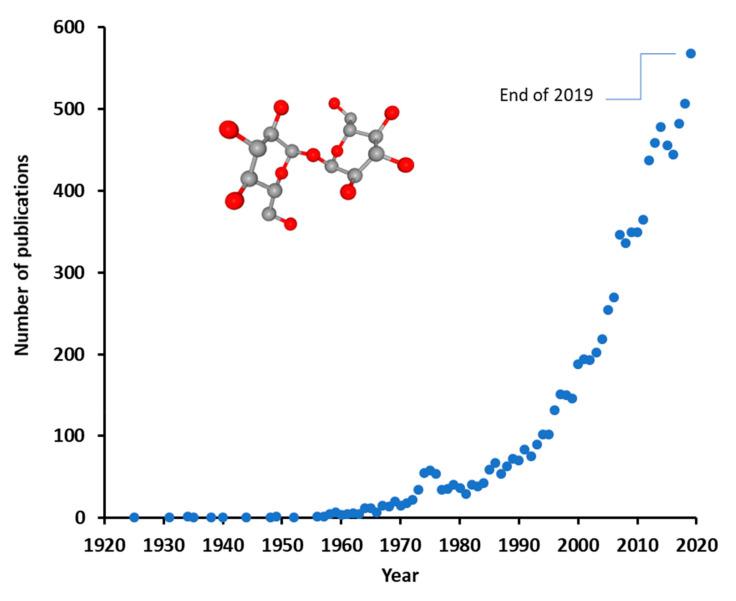
Annual number of publications with term “trehalose” in years 1925–2019 [1].

Trehalose is a disaccharide occurring naturally in various life forms, but not found in vertebrates. Trehalose is raising increasing interest for the development of various applications in food, cosmetic, and pharmaceutical industries, as reflected by almost an exponential growth in the accumulation of scientific publications (Figure 1). This interest may be explained by the proliferating body of evidence on the bioprotective characteristics of trehalose, its low toxicity, and proceedings in the development of more affordable production technologies.

Epithelial surfaces such as the cornea and the conjunctiva of the eye protect the underlying tissues against exogenous threats. Various environmental, intrinsic, or iatrogenic stressors may induce cell damage or cell death, leading to clinical diseases such as dry eye disease, also known as ocular surface disease (OSD). The detrimental processes are counteracted by several endogenous defense mechanisms.

The focus of the present review is to discuss the scientific evidence on the functions of trehalose in oxidative stress, protein clearance, and inflammation, and in animal models related to dry eye. The main objective is to help understand why trehalose is continuously showing beneficial effects in clinical trials in ophthalmology, especially in patients with OSD. The discussion is supplemented with patent data for the use of trehalose in dry eye and with some future prospects.

## 2. Chemistry and Sources of Trehalose

Since the early observation of sugar crystals isolated from solutions of the ergot of rye [2], trehalose (α,α-trehalose) has been found in numerous species of plants, fungi, algae, micro-organisms, insects, and other invertebrates, but not in mammals or other vertebrates, whereas α,β- and β,β-trehalose are almost absent in living organisms. Trehalose is a nonreducing disaccharide composed of two units of d-glucose with an α,α–1,1–glucosidic bond (Figure 1). This chemical structure has made trehalose known for its superior stability among all sugars. A historical account of trehalose from cultural links of the ancient “manna” to scientific research since Wiggers [2] has been given by Richards et al. [3] Natural occurrence and various applications of trehalose in food, health, and pharmaceutical industries have been presented by Richards et al. [3] and Cai et al. [4].

While not being synthesized in the human body, trehalose ingested from food is hydrolyzed into two D-glucose molecules in the small intestine by trehalase, a trehalose-specific disaccharidase. Trehalase has been found also in kidney, liver, and peripheral lymphocytes [3]. Despite substantial consumption and uses of trehalose by human beings, no toxic adverse effects have been reported, apart from low incidences of trehalose malabsorption [3].

The production of trehalose initially included chemical synthesis, microbial fermentation, and enzymatic and transgenic processes. However, the price of trehalose remained relatively high until the mid-1990s. A two-enzyme method was then developed, in which maltooligosyltrehalose synthase converts the α,α-1,4 bond of starch to α,α-1,1, and maltooligosyltrehalose trehalohydrolase releases trehalose by hydrolysis. This method and other (bio)synthetic routes for profitable large-scale industrial production of trehalose are discussed in detail by Ohtake & Wang [5].

## 3. Trehalose as a Bioprotectant

The bioprotective characteristics of trehalose have been demonstrated in several experimental models of wound healing and tissue injury. Relevant data were searched by using terms “Trehalose” [MeSH (Medical Subject Headings)] AND (“Wounds and Injuries/prevention and control” [MeSH] OR “Wounds and Injuries/therapy” [MeSH]) in PubMed. Found articles (11 results as of 13 May 2020) are briefly introduced to provide evidence that similar effects could be found in ocular models as well.

Animal studies by Cejková et al. [6,7,8] demonstrated the protecting effect of trehalose on the ultraviolet-B (UVB)-irradiated cornea (see Section 5.1). The reports conclude that trehalose protects ocular tissues from photodamage by supporting the viability and healing of the irradiated cornea and by suppressing hypoxic, oxidative, inflammatory, and apoptotic pathways of tissue damage.

Takahashi et al. [9] demonstrated that trehalose reduces neuronal damage in a spinal cord ischemia model in rabbits. Functional scores with 5% trehalose given as infusion or intravenously were significantly higher than in controls. Motor neurons were histologically normal with minimal inflammatory cell infiltration. Neither toxicity nor hemodynamic effects were observed. The authors postulate the protective effect to be related to preservation of cell membranes, mitochondria, and other cytoplasmic structures.

Traumatic brain injury and neurodegenerative disorders involve changes in brain concentrations of transition metals. Oral administration of 2% trehalose improved several cognitive outcomes impacted by brain injury [10] and induced a significant increase in brain zinc levels while not affecting iron and copper in aged mice [11], but not in young mice [10]. The authors also found that the expression of proteins involved in synaptic activity, neurogenesis, and neuroprotection was increased significantly in the brain after trehalose treatment, while maltose (another disaccharide) was inactive. These observations may have direct implications in ophthalmology because of the role of zinc in retina and cornea [12].

Lee et al. [13] reported a method to thermostabilize an enzyme with an activity for scar proteoglycans. When the trehalose-stabilized enzyme was administered by sustained topical release to rats with a spinal cord hemisection injury, digestion of astroglial scar proteoglycans, growth axons, and recovery of locomotor function were enhanced. The ability of trehalose to protect protein structures was also demonstrated in a study using trehalose in antibody-loaded nanoparticle emulsion [14]. Trehalose significantly improved the antagonist bioactivity of the antibody and protected it from denaturation during processing steps, notably during lyophilization, whereas its release kinetics were not affected, likely due to the small molecular size of trehalose. Protection of nanoparticles from freeze-drying (lyophilization) was reported also by Ruozi et al. [15]. Perfusion of partial-thickness wounds with 1% trehalose promoted healing and supported re-epithelialization and organizing of the tissue in a wound dressing model in pigs [16]. These findings imply that trehalose may be used as an excipient protectant in medicinal preparations comprising biological macromolecules or emulsion structures.

## 4. Inflammatory and Oxidative Stress Signaling

As a disaccharide, trehalose is usually considered impermeable to cell membranes, apparently leaving no chance to affect intracellular signaling. However, several studies have shown trehalose to pass to the cytosol of mammalian cells spontaneously in millimolar concentrations (reviewed in [17]). The suggested mechanism for this behavior is fluid-phase endocytosis that involves internalization of cell environment by vesicular “drinking” [18]. Moreover, trehalose traverses the plasma membrane via Solute Carrier Family 2 Member 8 (SLC2A8), a homolog of the trehalose transporter-1 (Tret1) [19]. By the simple presence of a high extracellular concentration of trehalose, the cell would take up trehalose that is conveyed to the lysosomal pathway. Due to its high chemical stability, trehalose is not degraded in lysosomes; therefore, it may leak from lysosomes into the cytoplasm [20]. There is evidence that the pH gradient changes the permeability of phospholipid bilayers of the lysosomal membrane and allows the leakage of trehalose and other low-molecular-weight molecules into the cytosol [20]. It is therefore reasonable to infer that the effects of trehalose on intracellular processes presented in this chapter are a result of trehalose acting directly in the cytosol. Representative and recent papers were obtained using PubMed search terms “Trehalose”[MeSH] AND “Anterior Eye Segment”[MeSH] (19 Dec 2019; Section 4.1), “trehalose” AND “oxidative stress” (16 Dec 2019; Section 4.2), “trehalose” AND “autophagy”, “trehalose” AND “proteasomes”, “trehalose” AND “inflammation” with term “eye” (16 Dec 2019; Section 4.3).

### 4.1. Trehalose in Ocular Surface Physiology

The ocular surface is constantly exposed to atmospheric oxygen and other environmental stressors, including various types of radiation, particulate or gaseous air pollutants, microbes, and desiccation. These stressors generate free radicals such as reactive oxygen species (ROS) that may damage corneal and conjunctival epithelia or disrupt corneal physiology and function. When cultured human corneal epithelial cells (hCEC) were subjected to desiccation stress for 5 to 45 min, trehalose-containing eye drops, unlike many other dry eye drops, showed high efficiency in maintaining normal cellular morphology, cell membrane function, and cell proliferative activity, and in preventing desiccation-induced cell death [21]. Combining trehalose with sodium hyaluronate in eye drops has been shown to elicit corneal re-epithelialization in response to corneal cross-linking compared with sodium hyaluronate alone [22]. Pretreatment with 3% trehalose alone preserved morphological and morphometric features of cornea in the laser subepithelial keratomileusis (LASEK) [23]. Trehalose has been observed to improve drug penetration from nanoparticles across the cornea, postulated to depend on pore formation during freeze-drying processing of the nanoparticles or the flexibility of hydrogen bonding between trehalose and the particles [24].

Ocular surface cells respond to environmental stress by increasing antioxidant and molecular chaperone production [6,25]. Moreover, the ubiquitin proteasome system (UPS) and lysosomal autophagic clearance are activated to clean damaged proteins [26]. Molecular chaperones (heat shock proteins, HSPs) are the only sensor system in cells with the ability to restore proteins to their original folding state, maintaining their function and preventing detrimental protein aggregation. HSPs are divided into different families according to their molecular weight and specialized cellular location. Interestingly, trehalose also functions as a molecular chaperone [27,28]. Similar to cytoplasmic HSPs, trehalose mediates its effects mainly via cytoplasmic connections. It is weakly known whether trehalose penetrates other organelles than lysosomes in cells. Lysosomal accumulation seems to be involved in endocytic cellular intake [17]. Once the molecular chaperone capacity responding to oxidative stress is exceeded, denatured proteins are degraded by the UPS. Usually, large protein aggregates and damaged cellular organelles undergo autophagic clearance [29]. Inflammatory signaling is activated as a host defense mechanism in acute stress, but the response is shifted to detrimental chronic inflammation in a prolonged or excessive stress conditions that may finally lead to cellular morphological and functional changes and to cell death.

### 4.2. Trehalose in Oxidative Stress Signaling

**Figure 2:**
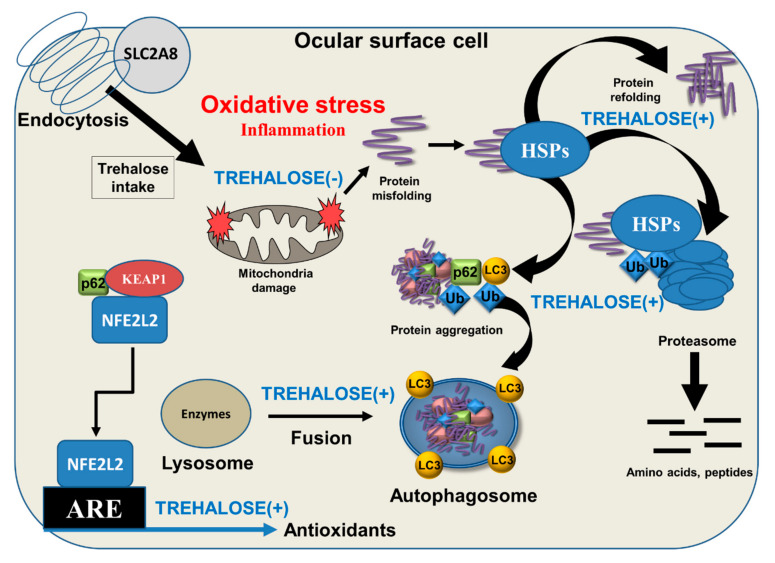
Schematic presentation of intracellular cytoprotective effects of trehalose. Trehalose intake to cells is via endocytosis and under the control of Solute Carrier Family 2 Member 8 (SLC2A8). Ocular surface cells are constantly exposed to environmental oxidative stress, which may evoke mitochondrial damage, protein misfolding, aggregation, and inflammation. Trehalose helps protein refolding together with heat shock proteins (HSPs) and enhances ubiquitin (Ub)-mediated proteasomal and microtubule-associated protein 1A/1B light chain 3 (LC3)-controlled autophagic clearance. LC3 and p62/SQSTM1 have binding sites to Ub that direct the sealed material to autophagic degradation. Moreover, p62/SQSTM1 regulates antioxidant production via transcription factor NFE2L2 released from NFE2L2-KEAP1 (kelch-like ECH-associated protein 1) and p62/SQSTM1 complex, allowing its binding to the antioxidant response element (ARE) protein and promote the transcription of antioxidative proteins. All beneficial functions of trehalose (TREHALOSE (+)) prevent oxidative-stress-induced inflammation (TREHALOSE (-)).

Oxidative stress is a consequence of the use of oxygen in aerobic respiration by living organisms, and it is denoted as a persistent condition of imbalance between the generation of ROS and the ability of the endogenous antioxidant system to detoxify them. Nuclear factor erythroid 2-related factor 2 (NFE2L2) is the key transcription factor in the sensing of oxidative stress. NFE2L2 was found to associate with the outer mitochondrial membrane and to protect mitochondria from oxidative damage, likely through direct interaction with mitochondria [30]. Upon activation, NFE2L2 is released from NFE2L2-KEAP1 (kelch-like ECH-associated protein 1) and the mitochondrial outer membrane serine/threonine protein phosphatase 5 complex, allowing for translocation of NFE2L2 from the cytosol into the nucleus and binding to the antioxidant response element (ARE); this promotes the transcription of more than 200 genes, including detoxification and antioxidant enzymes [31,32]. Trehalose has been shown to increase p62/SQSTM1 protein expression, activate NFE2L2, enhance the expression of its downstream antioxidant factors, and reduce the amount of ROS [33] (Figure 2). p62/SQSTM1 has a multifactorial role in acting as a regulatory protein between UPS and autophagy clearance as well as in the antioxidant response during oxidative stress [32,34]. Ischemic insult-induced protein aggregation was prevented by trehalose treatment via preservation of proteasome activity [35]. Benaroudj et al. [36] first documented that trehalose decreased the initial appearance of misfolded proteins presumably by scavenging free radicals. This observation was recently corroborated by Cejka et al. [8] who showed that trehalose decreases the amount of ROS, lipid peroxidation end-products, and DNA damage in UVB irradiation-exposed rabbit cornea.

### 4.3. Trehalose in Autophagy and Inflammation Signaling

Autophagy is an essential catabolic lysosomal mechanism that enables cells to clean damaged intracellular components [37]. It is a conserved process involving double-membrane autophagosomes to seal the cytoplasmic contents. Once autophagosomes fuse with lysosomes, lysosomal enzymes are released into the lumen of the autophagosome, leading to protein degradation. Autophagy enhances the clearance of toxic, cytoplasmic, aggregate-prone proteins and infectious agents. The beneficial roles of autophagy are linked to improved cell survival and to prevention of inflammation [38,39]. There is strong evidence that trehalose stimulates autophagy through the adenosine monophosphate-activated protein kinase (AMPK) independent of mechanistic target of rapamycin (mTOR) [40,41,42]. In addition to activating autophagy, trehalose preserves protein structural integrity and reduces aggregation of pathologically misfolded proteins (Figure 2). Impaired autophagy may evoke inflammation, which is associated with OSD [43]. Trehalose induces autophagy and reduces secretion of cytokines IL(interleukin)-6, IL-8, and monocyte chemoattractant protein-1 (MCP-1) in corneal cells under tumor necrosis factor-α (TNF-α) and desiccation stress. Lipopolysaccharide-induced secretion of IL-1β, IL-6, TNF-α, and nitric oxide are decreased in trehalose-treated cell cultures [44]. In addition, trehalose suppresses microglial activation via transcription factors nuclear factor-κB (NF-κB) and activating protein-1 (AP-1). When lysosomal enzyme activity is disturbed, trehalose may prevent cellular damage and suppress neuroinflammation [45]. Local inflammation linked to peripheral nerve injury is an important feature in dry eye [46].

## 5. Trehalose in Ophthalmic Applications

### 5.1. Animal Models

Publications describing the effects of trehalose in various ocular animal models were searched in PubMed by using terms “Trehalose” [MeSH] AND “Eye” [MeSH] AND “animals” [MeSH Terms:noexp] (13 May 2020). Some of the found papers concern the use of trehalose as an adjuvant for cryoprotection and in other cellular systems [24,47,48,49,50], or the administration by oral route [45]. These reports are not directly within the scope of this chapter, but they corroborate the findings by others in that trehalose is safe to various cell types and supports the biological and morphological properties of tissues in high (up to 35%) [47] concentrations.

Chen et al. [51] used an experimental model of dry eye in BALB/c mice. After being housed in a controlled environment with low humidity and constant airflow and temperature for 21 days, the animals were randomized for treatment with phosphate-buffered saline (PBS) control, 3% (87.6 mM) anhydrous trehalose, or pooled mouse serum eye drops (10 µL) administered every 6 h in the same environment for the next 14 days. The housing conditions significantly reduced tear production from 2.3 to 1.7 mm by phenol thread wetting test. Trehalose and serum eye drops increased tear secretion significantly and similarly to 1.9 and 2.0 mm, respectively, at 14 days, but not yet at seven days, while control treatment showed no improvement. In an analogous fashion, the desiccating environment induced corneal fluorescein staining from 1.6 to 9.8 score units (on scale 0–15) at day 21; trehalose and serum treatments decreased staining scores to 6.6 and 4.5 units, respectively, at 14 days that was not seen in PBS controls. Trehalose also increased the thickness of corneal epithelium and the number of goblet cells, and, as expected, decreased the number of ruffling, desquamating, and active caspase-3-positive apoptotic cells on the ocular surface epithelium; all changes were improvements from the adverse effects induced by the dry environment. The authors concluded that the data was consistent with previous reports providing evidence on the role of apoptosis in dry eye pathogenesis.

Li et al. [52] used the controlled environment system described by Chen et al. [51] for a different mouse strain. After being housed in the controlled dry environment for 21 days, C57BL/6 mice were randomized for groups of untreated controls, treatment with PBS, or with 3% anhydrous trehalose eye drops (10 µL) every 6 h in the same environment for another three weeks. Trehalose significantly restored corneal epithelial integrity that was impaired in the controlled environment, as graded by two masked observers. Corneal epithelial occludin staining, indicating integrity of corneal epithelial barrier and tight junctions, was more homogenous in the trehalose group. Cell desquamating was absent, and expression of involucrin and small proline-rich protein 2 was at normal level with trehalose. Similarly, corneal epithelial expression levels of HSP70 and matrix metalloproteinase (MMP)-9 increased markedly in response to desiccation but returned to normal levels in trehalose-treated eyes. Conjunctival IL-1β, IL-2, IL-6, IL-17, TNF-α, and MMP-9 mRNA expression was also lower with trehalose than in control groups. The authors concluded that trehalose restored ocular surface integrity, suppressed the expression of inflammatory and proteolytic factors and keratinization in eyes exposed to dry environment.

Four studies on the protecting effect of trehalose in a UVB-irradiation-induced ocular damage model have been published by Cejková and coauthors. In the first study [25], the eyes of anesthetized adult New Zealand white rabbits were exposed to a daily dose of 0.5 J/cm^2^ UVB irradiation (spectral intensity peaking at 312 nm) for four days. During irradiation and at three other times during the day, 3% (87.6 mM) anhydrous trehalose eye drops were instilled on one eye and saline control on the other eye. Central corneal thickness, used as a measure of corneal hydration in live animals by ultrasonic pachymeter, showed less increase with trehalose than with control drops on day 4. Trehalose treatment reduced vascularisation and the number of inflammatory cells in excised corneas obtained from sacrificed animals on day 5, compared to saline. Corneal transmittance in UV and visible wavelength regions (about 290–570 nm measured) was greatly impaired after UVB treatments and partially prevented by trehalose in comparison to control. UVB-induced apoptosis, immunohistochemically detected as active caspase-3 staining of the corneal stroma, was significantly reduced or even absent in eyes treated with trehalose. Trehalose also decreased the corneal expression of nitric oxide synthase and nitrotyrosine, a toxic reaction product of nitric oxide. The authors concluded that trehalose reduced both UVB-induced damage caused by reactive oxygen and nitrogen species and their adverse effects on corneal optics.

In a test protocol similar to the previous study, Cejková et al. [6] analyzed the excised rabbit corneas immunohistochemically. The NFE2L2-regulated expression of the antioxidant enzymes catalase, glutathione peroxidase, and superoxide dismutase decreased during UVB irradiation with saline eye drop treatment, whereas their expression intensities were maintained close to the initial levels with 3% trehalose. The expression of pro-oxidant enzyme xanthine oxidase increased during UVB irradiation with saline and was maintained at normal level with trehalose. The expression of proinflammatory cytokines IL-6 and IL-8 were almost absent at baseline, increased by UVB, and reduced by trehalose during irradiation. HSP70 and MMP-9 expression followed a similar pattern. The authors concluded that trehalose strongly protected the UVB-irradiated cornea against the development of antioxidant/pro-oxidant imbalance, a condition where oxidative damage to the cornea takes place due to insufficient depletion of reactive oxygen species.

Čejková et al. [7] further demonstrated that, in addition to corneal oxidative damage, the UVB-irradiated cornea also suffers from hypoxia due to the inability of the damaged corneal cells to utilize oxygen normally. Hypoxic conditions delay re-epithelialization, the ingrowth of vessels into the cornea, and apoptotic cell death. In this study, trehalose, applied on the surface of corneas during a daily UVB irradiation dose of 0.5 J/cm^2^ at 312 nm for two weeks, improved corneal healing and transparency and suppressed corneal neovascularization. Suppression of apoptotic cell death, as detected by expression of active caspase-3, was evident after one week, and the expression of nitrotyrosine, malondialdehyde, and urokinase-type plasminogen activator returned to normal levels during two weeks of trehalose treatment. The authors’ conclusion was that trehalose accelerated the healing of the UVB-irradiated cornea very probably via suppression of hypoxic injury.

In the fourth study [8], the authors believe to be the first to demonstrate that trehalose reduces excessive ROS in the UVB-irradiated cornea. In one set of experiments, New Zealand white rabbits were exposed to a daily dose of 0.5 J/cm^2^ UVB irradiation and simultaneously treated six times daily with 3% or 6% trehalose eye drops for four days. ROS production, oxidative stress, and DNA damage were reduced in corneas treated with 3% trehalose and more efficiently with 6% trehalose. In another set of experiments, the animals were irradiated for four days and thereafter treated with trehalose for 14 days. Both eye drops healed oxidative injuries, restored corneal transparency, suppressed corneal neovascularization, and decreased corneal thickness. The higher concentration was again more effective in these processes.

### 5.2. Clinical Pilot Studies in Human Subjects

In a study aiming to evaluate the effect of trehalose on the corneal epithelium in LASEK procedure, Aragona et al. [23] recruited 12 patients (five females, seven males) undergoing photorefractive keratectomy to a controlled nonrandomized study. The patients received 3% trehalose (Thealoz^®^, Laboratoires Théa, Clermont-Ferrand, France) in their right eye, followed by 0.4% oxybuprocaine hydrochloride in both eyes 5 min later. The vitality of epithelial flaps was significantly increased in samples taken from trehalose-treated eyes. No apoptotic cells were observed in either eye. Statistically significant differences were found for a number of morphometric parameters, including corneal epithelial thickness, basal cell area, optical cytoplasmic density, and the distribution of desmosomes and hemidesmosomes. Trehalose was concluded to be able to preserve the morphological and morphometric features of the cornea during alcohol delamination used to expose the stroma for keratectomy. Ethanol is known to replace intracellular water, and the authors deduce trehalose to substitute water molecules and form hydrogen bonds that stabilize protein structure and preserve the physiological morphology of epithelial cells.

Fariselli et al. [53] reported the results of an open-label and uncontrolled pilot study in 15 patients (including 14 females) diagnosed with evaporative dry eye (Ocular Surface Disease Index (OSDI) > 18, Schirmer test > 10 mm/5 min, tear film break-up time (TBUT) < 10 s, National Eye Institute/Industry grading (NEI) score > 3). For an initial wash-out period of two days, sterile saline solution was instilled in both eyes. Baseline results obtained before and after wash-out were combined. Patients administered one drop of Thealoz^®^ Duo (Laboratoires Théa, Clermont-Ferrand, France) in both eyes three times a day for two months. All enrolled participants completed the study. For OSDI, 14 out of the 15 patients reached a reduction of at least 20% set as a target outcome; a significant reduction of the mean OSDI score from about 39 to 28 units in a month and to about 23 units in two months was observed. Symptom intensity measured by Visual Analogue Scale (VAS) scoring reduced accordingly. Statistically significant reductions in vision-related functions and environmental triggers subscales rather than in ocular symptoms subscales of OSDI were observed. Significant improvement was achieved in TBUT, corneal and conjunctival damage scores, and goblet cell density at the two-month endpoint, whereas the increase in MUC4 mucin expression did not reach significance. Cytokines IL-1β, IL-6, and IL-8 in tear fluid correlated to surface damage parameters at baseline and showed a significant decrease at endpoint of two months.

The combination of 3% trehalose and 0.15% hyaluronic acid (HA) eye drops (Thealoz^®^ Duo) were compared with 0.15% HA (Eye Still^®^; Teka, Istanbul, Turkey) for treatment of corneal cross-linking and epithelial healing [22]. Both eyes of 23 patients underwent epithelium-off corneal cross-linking in two separate sessions. The first operated eye of each patient was treated with HA eye drops alone six times a day until complete re-epithelialization. After about two weeks, the second eye was operated and treated similarly with trehalose-HA eye drops until re-epithelialization. The observed mean corneal epithelial healing time was 2.3 days for trehalose-HA and 3.8 days for HA eye drops with significant difference, suggesting faster corneal re-epithelialization with trehalose.

### 5.3. Randomized Controlled Trials

Evidence for the benefits and risks of trehalose in ophthalmologic applications including treatment of dry eye should be presented in randomized controlled trials. The best approach for collecting all relevant data for this chapter was found using search terms “trehalose” and “eye” in PubMed. From among the 52 publications (27 Mar 2020), less than 20 articles fulfilling the requirement for a randomized controlled trial were selected and allocated into subsections for dry eye, ocular surgery, and tear film dynamics. The level of evidence addressed to each dataset was adapted from the modified American Academy of Ophthalmology Preferred Practices guidelines [54]. The main details of each study are summarized in Table 1.

#### 5.3.1. Clinical Trials on Dry Eye

Matsuo et al. [55] was the first clinical investigation on the use of trehalose eye drops in patients with dry eye and the only study comparing at least two trehalose concentrations (Table 1). The authors documented data from 34 Japanese patients, 18 of whom were randomized to use eye drops containing 100 mM trehalose (corresponding to 3.4%, *w*/*v*, of anhydrous trehalose; actual hydration state was not disclosed) in saline vehicle on one eye and saline control on the fellow eye six times daily for four weeks. As a comparator group, 16 patients used 200 mM (6.8%) trehalose and saline, respectively. The selected patients manifested moderate to severe dry eye and seropositivity to Sjögren syndrome autoantibodies; the study population thus consisted of autoimmune patients. At weeks 2 and 4, dry eye symptom scores did not significantly improve with 100 or 200 mM trehalose treatment compared with saline. TBUT increased significantly in eyes with 100 mM trehalose, but not with 200 mM trehalose compared with saline. Staining scores improved significantly with both concentrations. No adverse effects were observed. The authors point out that the osmolarities of the 100 and 200 mM trehalose drops were 1.3 and 1.8 times that of isotonic saline, respectively, i.e., hyperosmolar. Despite the lack of statistical significance, the authors stated that both trehalose treatments were effective overall in improving symptoms and signs of dry eye. In respect to TBUT, they conclude the 100 mM concentration to be a better dose.

In a further trial by Matsuo et al. [56], mostly female patients with moderate to severe dry eye were enrolled, 64% with a diagnosis of Sjögren syndrome, and 81% complaining of dry mouth (Table 1). Two four-week treatment periods followed a cross-over design without a wash-out phase. The nonrandomized assignment of patients into the control treatment with either 0.1% HA (up to four weeks) or hydroxyethyl cellulose eye drops (up to eight weeks) was based on “arbitrary” considerations. Before the start of the trial, five subjects had already used 100 mM trehalose while three other subjects had used 200 mM trehalose for six months as participants in the previous trial [55] (Table 1); they were allowed to continue using the same concentration in both eyes. The order of cross-over periods was randomized, and the eye drop bottles were masked. Both control formulations contained benzalkonium chloride, whereas the 100 mM trehalose drops in saline were unpreserved. The drops were administered in both eyes four times a day for four weeks. Symptom scores did not differ at week 4, but they improved more significantly with trehalose than with hydroxyethyl cellulose at week 8. TBUT increased and staining scores improved significantly with trehalose. A majority of the patients considered trehalose superior to HA or hydroxyethyl cellulose in treating the symptoms.

After the initial studies by Matsuo et al., clinical trials have been continued using commercial products containing trehalose. Pinto-Bonilla et al. [57] claim to be the first to study eyedrops containing both trehalose and HA (Table 1). In this open-label cross-over trial, 3% trehalose and 0.15% HA (Thealoz^®^ Duo) was used five times a day for seven days in comparison to Systane eye drops, a preserved formulation with hydroxypropyl guar, polyethylene glycol, and propylene glycol. The washout period was five days. No blinding was used. Seventeen patients with moderate or severe dry eye (OSDI >25) were recruited, randomized, and included in efficacy and safety populations. Study diary-based patient global satisfaction, dry eye symptoms, and their impact at work improved significantly more with Thealoz^®^ Duo than with Systane. OSDI score improved more with Thealoz^®^ Duo, but without statistically significant difference. Ocular staining scores improved with both treatments. More patients preferred Thealoz^®^ Duo than Systane. No adverse events were reported. The authors recognized the study to be a small one and with relatively short duration. The study was sponsored, and the preparation of the paper was remunerated by Théa.

Chiambaretta et al. [58] conducted an investigator-masked multicenter study in a total of 105 adult patients with moderate to severe dry eye (OSDI ≥ 18 and with either Schirmer test 3–9 mm/5 min or the sum of three TBUT ≤ 30 s in at least one eye) (Table 1). Fifty-two patients used one drop of Thealoz^®^ Duo (3% trehalose and 0.15% HA in hypo-osmolar formulation) 3–6 times a day for 84 days. All patients in this group completed the study. Fifty-three patients received control eye drops (0.18% HA, also hypo-osmolar; Vismed^®^, Horus Pharma, Saint-Laurent du Var, France). Of these, 46 and 45 patients, respectively, completed the study per protocol. Noninferiority of Thealoz^®^ Duo to HA alone, assessed by Oxford grading score to assess global severity of ocular keratitis and conjunctival impairment, was demonstrated at day 35. Subjective scores evaluated by patients or investigators improved more with Thealoz^®^ Duo. Schirmer test, TBUT, conjunctival hyperemia, and global performance assessed as secondary efficacy criteria were similarly improved in both groups with no clinically meaningful differences. Both treatments were well tolerated. Fewer ocular symptoms and fewer adverse events occurred with Thealoz^®^ Duo. The authors noted that the primary criterion was assessed by investigators masked to treatments, limiting open-label bias. They concluded that the HA–trehalose combination treatment is effective and safe, with better patient satisfaction, than existing HA eyedrops, particularly from the first month of treatment. The study was supported by Théa.

The study by Doan et al. [59] was a post hoc analysis of the Chiambaretta study data [58] (Table 1). The authors reanalyzed the percentage of patients with OSDI score below and above the threshold value 19 at study endpoint days 35 and 84 after daily HA–trehalose and HA treatments. There were more patients with OSDI < 19 in the HA–trehalose group (46.2%) than in the HA group (32.1%) at day 35; the difference was nonsignificant. At day 84, the proportions were 78.8% and 58.5%, respectively, with a significant difference. The authors acknowledged the lack of blinding of the patients to treatments as a limitation, but supported the conclusions of Chiambaretta et al. on symptomatic relief of dry eye with HA–trehalose eyedrops.

An observer-masked, two-way cross-over study by Fondi et al. [60] investigated the clinical effect of a gel product containing 3% trehalose, 0.15% HA, and 0.25% carbomer and sorbitol (THC gel; Thealoz^®^ Duo Gel) compared with HA–trehalose (Thealoz^®^ Duo) (Table 1). Forty-five patients with moderate to severe dry eye (OSDI ≥ 22 and TBUT ≤ 10 s or Schirmer I test 2–5 mm/5 min) were enrolled, out of whom 40 completed the study per protocol. The worst eye was selected as the study eye. During a one-week washout period before randomization, all participants used HA eye drops (Hyabak^®^, Laboratoires Théa, Clermont-Ferrand, France) until three days before the start of the study, then used saline drops (Hydrabak, Théa) until 12 h before the first study day. The randomized treatments were used for one week as frequently as needed (pro re nata) during the day. In addition, all patients in both groups were asked to instill the THC gel before going to sleep. The washout period was one week. TBUT and subjective VAS scoring for sleep quality increased while staining scores and OSDI decreased with nonsignificant differences between treatments. Schirmer I test scores and safety parameters did not change. As the mean frequency of instillation was 3.2 times/day for HA–trehalose eye drops and 1.9 times/day for THC gel, the authors concluded that that the THC gel treatment requires less frequent instillation to achieve a comparable benefit to the patient. The study was sponsored by Théa.

Panigrahi et al. [43] compared eye drops with 3% trehalose and 0.1% HA (Trehalube^TM^, Micro Labs, Bangalore, India; A. Ghosh, personal communication) to eye drops with 0.5% carboxymethylcellulose (CMC; Lubrex^®^, Micro Labs, Bangalore, India), both preserved with an oxychloro complex, in nine patients with mild dry eye signs, but with moderate to severe symptoms (OSDI > 23). The eye drops were administrated in a patient-blinded fashion (Ghosh, personal communication) twice a day in randomized contralateral eyes for 30 days. Mean OSDI (measured separately for both eyes) reduced and TBUT increased significantly with trehalose. Tear secretion was not affected. Trehalose also reduced proinflammatory cytokine levels (Table 1).

In a clinical trial by Laihia et al. [61], 2% trehalose and 0.2% high-molecular-weight (1.0–1.5 MDa) HA were included as active components in a hypotonic multi-ingredient microemulsion eye drop formulation (Table 1). Isotonic eye drops with 0.2% medium-molecular-weight (0.7–0.9 MDa) HA were an active control treatment. Both eye drops were preservative-free solutions and masked at all levels. The study comprised three parts. In the third part, 26 randomized participants used the same eye drops for both eyes three times a day for 30 days. Statistically significant improvement with HA–trehalose microemulsion eye drops was observed in TBUT, ocular protection index (=TBUT/interblink interval), corneal and nasal conjunctival staining, conjunctival and lid redness, and OSDI. Tear osmolarity in initially hyperosmolar (≥ 308 mOsm/L) subjects decreased similarly to healthy level with both treatments, suggesting an insignificant role for solution tonicity. A significant difference between treatments was achieved with ocular protection index. The authors hypothesize that disrupting hyperosmolar stress would lead the way to biophysical protection of the ocular surface to resolve epithelial damage and inflammation, followed by relief of symptoms. A multi-ingredient formulation could be more efficient against all etiologic factors of dry eye [70,71]. The authors point out that the measurements were performed many hours after the last eye drop’s instillation. The role of trehalose was likely not a short-term increase in tear film thickness (TFT) as observed previously [66,67] (see 5.3.3), but rather antioxidative, cytoprotective, and wound-healing activity on the ocular epithelium. The study was sponsored by Finnsusp, the manufacturer of both investigational eye drops.

Downie et al. [62] compared nanoemulsion eye drops (OM3) containing CMC, glycerol, flaxseed (linseed) oil, castor oil, levocarnitine, erythritol, and trehalose (Refresh Optive^®^ MEGA-3, Allergan, Dublin, Ireland) to a control vehicle without trehalose and flaxseed oil (Table 1) labelled as inactive ingredients in the product. Patients with stratified dry eye severities not exceeding an OSDI score of 65 were randomized to use OM3 (n = 120) or control eye drops (n = 122) in preservative-free and double-masked single-unit dose vials at least twice a day for 90 days. Significant improvement from baseline was achieved in OSDI, TBUT, and corneal and conjunctival staining at each visit (days 7, 30, 60, and 90) with OM3. OM3 was found to be noninferior to control eye drops in reducing symptom severity at 90 days. Both treatments were well tolerated. The authors interpret trehalose and flaxseed oil to offer additional protection to the ocular surface, although distinguishing the relative benefit of the two ingredients was not possible. The study was funded by Allergan, the manufacturer of both investigational eye drops; all authors reported a prior or current financial or personal relationship with the company.

#### 5.3.2. Clinical Trials on Ocular Surgery

A controlled investigator-blinded study evaluated the usability of trehalose for laser-assisted in situ keratomileusis (LASIK) post-treatment [63] (Table 1). Thirteen enrolled patients (12 males) undergoing sequential bilateral LASIK surgery were randomized into two treatment groups. In group 1 (n = 6), 0.15% HA eye drops (Hyabak, Théa) were taken every two hours during the first 10 days and six times a day thereafter until three months after surgery. In group 2 (n = 7), eye drops with 3% trehalose (Thealoz^®^, Théa; no HA) were administered four times a day, each time followed by HA (Hyabak) eye drops five minutes later and starting three days before surgery. Ocular assessments were performed on days 1, 7, 30, and 90. In addition, frequent and gradually reducing doses of dexamethasone and tobramycin were administered for 10 days after surgery. Data corrected for presurgery values showed no significant differences between treatments in OSDI, tear osmolarity, and TBUT, while the Symptom Assessment in Dry Eye (SANDE) scoring on an analogue scale showed significant improvement in symptom severity with trehalose at all study visits after surgery. Vital staining data (Oxford and NEI scales) revealed superiority of trehalose treatment with significant difference to HA on days 30 and 90. The authors postulate that the interaction of trehalose with ocular membrane lipids could provide additional protection against surgical trauma and increased osmolarity, resulting in reduced inflammation and cell death. The authors conclude that the recovery of tear and cell homeostasis after LASIK was superior for 3% trehalose compared to HA. The study was partially supported by Théa.

Caretti et al. [64] investigated the treatment of eyes after cataract surgery with THC gel (Thealoz^®^ Duo Gel) or with 0.15% HA (Hyabak^®^, Théa; La Gloria Valerio, personal communication) eye drops in a randomized case-control study (Table 1). Sixty eyes of 60 patients with mild to severe dry eye symptoms and scheduled for unilateral cataract surgery were randomized into two groups. After surgery, 30 eyes were treated with THC and 30 eyes with HA in a double-masked manner twice a day for one month. Steroid antibiotics and nonsteroidal anti-inflammatory drugs were given as well. All parameters were assessed at baseline and at 7 and 30 days after surgery. In the trehalose group, mean TBUT improved significantly from about 3.5 s to 6.3 s at day 7 and to 6.7 s at day 30; the corresponding values for HA were 4.1, 4.8, and 5.1 s, with significant difference between treatments. The mean OSDI score decreased with THC treatment from a preoperative level of 31 to 5 units and with HA from 21 to 11 units on day 30, showing a significant difference between treatments. Fluorescein staining (Oxford scale) and postoperative visual acuity improved with both treatments without statistically significant differences between treatments. Tear production did not show significant changes. Patients’ global satisfaction score was significantly greater for THC gel. It was concluded that THC was effective and well tolerated in reducing dry eye symptoms and in improving the clinical outcome after cataract surgery.

The efficacy of topical chloramphenicol 0.5%–betamethasone 0.2% (CB; Betabioptal^®^, Laboratoires Théa, Clermont-Ferrand, France) with and without THC gel (Thealoz^®^ Duo Gel) treatment following strabismus surgery was compared by Vagge et al. [65] (Table 1). This single-arm study involved 31 patients undergoing bilateral strabismus surgery. After surgery, the contralateral eyes were randomized to receive either topical CB alone or in combination with THC gel, instilled three times a day for four weeks. All patients received both treatments without masking. Conjunctival redness (Efron scale) decreased, but did not differ between eyes at 1 and 4 weeks after surgery. Subjective numerical ratings for foreign body sensation, burning or stinging, and stick feeling were significantly lower in the THC-treated eyes at week 1, but not at week 4, whereas blurred vision was rated significantly higher for THC at both time points. The authors interpreted that CB with THC gel was overall more effective than CB alone in reducing subjective symptoms of ocular discomfort after strabismus surgery, whereas the treatments are equally effective in reducing conjunctival inflammation.

#### 5.3.3. Clinical Trials on Tear Film Dynamics

The effect of trehalose on tear film dynamics was investigated in two studies utilizing a custom-built optical coherence tomography (OCT) system to measure TFT. The study by Schmidl et al. [66] involved 60 completing patients with mild or moderate dry eye (OSDI 13–32, TBUT ≤10 s and Schirmer I test 2–5 mm, 43 females and 17 males) (Table 1). After measurement of the baseline TFT, the study subjects received randomized and double-masked instillations of single doses of 3% trehalose and 0.15% HA (Thealoz^®^ Duo) or 0.15% HA (Hyabak^®^) or NaCl (Hydrabak^®^; all from Théa) in a parallel-group design in the worst eye only. TFT was measured at 10, 20, 40, 60, 120, and 240 min after instillation. TFT increased significantly from a baseline of 2.4 ± 0.4 (mean, SD) to a peak of 3.1 ± 0.9 µm at 10 min with HA–trehalose and from 2.4 ± 0.3 to 2.9 ± 0.5 µm with HA, followed by a gradual decrease up to 240 min. NaCl showed a negligible effect. A statistically significant difference in the time course between treatments was obtained (repeated-measures analysis of variance model). The trehalose–HA combination showed a significantly higher TFT at all time points, although the difference at 240 min was only a few per cent from baseline. All treatments slightly increased TBUT and Schirmer I score measured at 240 min only (*p* > 0.05), attributed by the authors to single dosing. Tolerability was similar with all eye drops. The authors conclude that trehalose increases ocular residence time of HA eye drops by an unknown mechanism that requires further studies to resolve. The study received an unrestricted grant from Théa and was used for a joint patent application (see Section 5.5).

The same research group conducted a similar OCT investigation of TFT using gel-based lubricants [67] (Table 1). In this study, sixty completing patients with moderate or severe dry eye (OSDI ≥ 22, TBUT ≤ 10 s and Schirmer I test 2–5 mm, 23 females and 37 males) received randomized and observer-masked instillations of single doses in both eyes in a parallel-group design. TFT was measured at 10, 30, 60, 120, 240, and 360 min after instillation. Baseline TFT was 3.53 ± 0.73 µm, a value remarkably higher than those reported [66] using the same OCT system. Peaking again at 10 min, Systane Gel (0.4% polyethylene glycol, 0.3% propylene glycol, hydroxypropyl guar and sorbitol; Alcon Pharma, Fort Worth, TX, USA) showed the greatest TFT increase to a mean of about 9 µm (+156% from baseline), followed by THC gel (Thealoz^®^ Duo Gel) and Hylo-Gel (0.2% HA and sorbitol; Ursapharm, Saarbrücken, Germany) with mean peak values of about 6 µm (+66%) and 5 µm (+33%), respectively. A relative increase of less than 20% from baseline at 60 and 120 min was significant for THC gel only. No significant differences were observed at 30 min after instillations. Overall, TBUT increased significantly and similarly at 6 h in all treatment groups. Schirmer I score did not change. The authors postulate that the polymeric meshwork created by cross-linked hydroxypropyl guar of Systane Gel may stabilize the ocular tear film and thus partially explain the increase in TFT compared to the other two formulations. In spite of this difference, they conclude that THC gel offers a longer residence time on the cornea. This study was sponsored by Théa.

In a more recent study, Karaca et al. [68] enrolled 122 patients with mild to moderate dry eye to investigate tear meniscus height (TMH) and depth (TMD), tear osmolarity, ocular residence time, and subjective comfort after single-drop administration of eye drops with THC gel (Thealoz^®^ Duo Gel) or 0.3% HA (Vismed^®^ Gel, TRB Chemedica, UK) (Table 1). For this randomized and observer-blinded study, only patients diagnosed with primary Sjögren syndrome and with OSDI score 13–32 units were included. The study eye was the one with the lower TBUT. After instillation of one drop of the randomized eye drops by one ophthalmologist, another ophthalmologist, blinded to study treatments (E. Karaca, personal communication), measured TMH and TMD using swept-source OCT for up to 240 min, interpreted to represent the corneal residence time of the eye drops. A significant increase was observed in TMH at 10 min and in TMD at 10 and 60 min with both treatments. At each time point for up to 120 min, TMH and TMD were significantly higher for 0.3% HA than for THC gel. TBUT, tear osmolarity, and Schirmer I test measured at 240 min did not differ from baseline, interpreted by the authors to reflect the single-dose treatment. The 0.3% HA eye drops were concluded to be superior to THC gel drops in terms of corneal residence time and patient comfort duration, although longer observation periods would be required to confirm this.

For understanding ocular residence time, it might be useful to note two nonclinical reports that discuss the importance of physical and rheological properties of HA and trehalose in eye drops. White et al. [72] developed a “comfort agent index” to facilitate direct comparison between different polymer agents used in eye drops and to provide experimental validation and explanation for general trends suggested by the available clinical data. Firstly, the main finding was that the comfort-promoting properties relate strongly to both the concentration and the molecular weight (i.e., chain length) of each agent. Secondly, polysaccharides as a group showed the best comfort agent index values, followed by acrylic agents. The greatest comfort property contributions, independent of specific molecular weight and concentration considerations, were from HA, hydroxypropyl methylcellulose, and CMC. In accordance with the main findings, not only the concentration, but especially the molecular weight of HA strongly contributed to the comfort property, as suggested by several previous studies in vitro [73,74,75,76]. This finding leads to the second paper by Salzillo et al. [77] who analyzed the molecular weights of HA and the viscosity of some eye drop formulations on the market. One of the analyzed brands was Hyabak^®^ (Théa), containing 0.15% HA and used in the TFT study of Schmidl et al. [66]. The HA molecular weight in this product was found to be 0.36 MDa, while the viscosity of the solution was 2.3 mPa s. As compared to another product containing 0.28% of 1.1 MDa HA and with a zero-shear viscosity of 24 mPa s, the remarkably low viscosity of Hyabak^®^ is obviously attributable to the low molecular weight of HA. Out of six products analyzed by Salzillo et al., Hyabak^®^ showed both the lowest HA molecular weight and the lowest viscosity. The HA concentration on the product label is the same for Hyabak^®^ and Thealoz^®^ Duo, and both product versions also utilize the same ABAK eye drop dispenser technology with a sterile filter membrane, suggesting that the HA species used in both Hyabak^®^ and Thealoz^®^ Duo is probably of the same low molecular weight. In a more recent analysis, the HA molecular weight in Thealoz^®^ Duo was confirmed to be 0.22 MDa and the viscosity 2.8 mPa s [78]. In an early study [79] cited both by Schmidl et al. [66] and Wozniak et al. [67], ocular residence time was 11.1 min for 0.2% HA and 23.5 min for 0.3% HA; thus, increasing HA concentration by 50% resulted in more than 100% longer residence time in real-life conditions. The results of these studies suggest that the observed ocular residence times [66,67] would be enhanced efficiently by increasing the concentration and/or the molecular weight of HA, as demonstrated by Karaca et al. [68].

**Figure 3:**
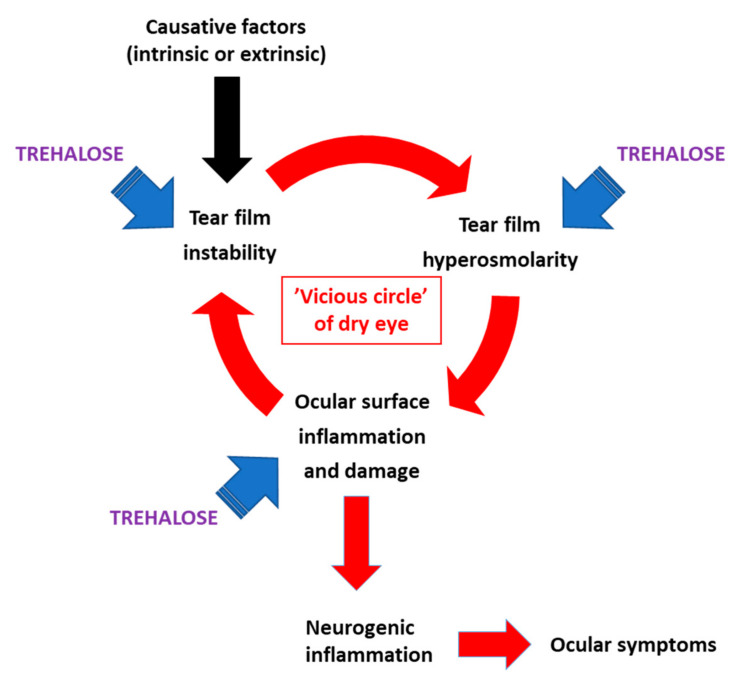
Etiologic factors of dry eye affected by trehalose as demonstrated in clinical data. Redrawn from ref. [61], Figure S1, with permission from the authors.

The pathophysiologic mechanism of dry eye, as recently defined by the TFOS DEWS II consortium [70,71], involves etiologic factors that maintain a “vicious circle” on the ocular surface, including tear film instability, accompanied by localized tear film hyperosmolarity, causing epithelial damage and inflammation. Each etiologic factor can be measured as an independent endpoint in clinical trials. The evidence above, collected from randomized controlled trials and supported by pilot studies in human subjects and animals, strongly suggests that trehalose can directly improve the condition of the affected eyes by obstructing the dry eye “vicious circle” at multiple points (Figure 3). This effect will help reach the healthy tear film homeostasis and minimize chances for a return to the adverse condition, defined as the goal of dry eye management [54].

### 5.4. Do We Know the Optimum Concentration?

The efficacy and safety of trehalose in adverse ocular conditions have been well established, but how was a profitable trehalose dose determined and who made that decision? Reviewing the scientific literature reveals that the “knowledge” of the “right” concentration obviously comes from one source. Matsuo et al. [55] is the first clinical investigation and to date the only report comparing at least two trehalose concentration levels in human subjects. The authors explain the concentrations 100 mM (3.4%) and 200 mM (6.8%; calculated as anhydrous trehalose) were based on their earlier preclinical work [80] in which hCEC cultures were shortly pretreated with trehalose and let dry at low humidity. Trehalose preincubation was found to protect cell viability significantly in concentrations 50 mM (1.7%), 100 mM (3.4%), and 200 mM (6.8%), compared with concentrations 20 mM or lower, maltose, buffer vehicle, hydroxyethyl cellulose eye drops, or 0.1% HA. The average results for 50 and 100 mM concentrations were practically identical (20.1% and 19.1% dead cells, respectively, with large deviations), while 200 mM trehalose was clearly more efficient (11.9% dead cells). Interestingly, however, Matsuo et al. [55] then interpreted those results to show that the 50 mM concentration was “less effective” and acknowledged the two highest concentrations as applicable for their clinical study. Considering the close relationship of the main author with Hayashibara Biochemical Laboratories, a company holding manufacturing and other patents for trehalose (see disclosure [55] and Section 5.5), there may be a commercial bias in the selection of concentrations. To our knowledge, scientific papers have remained silent about trehalose concentrations in eye drops since.

Consequently, almost all clinical trials with trehalose in dry eye patients since Matsuo’s reports have been conducted at the 3% concentration level (Section 5.2 and Section 5.3). Laboratoires Théa adopted this concentration for Thealoz^®^, Thealoz^®^ Duo, and Thealoz^®^ Duo Gel. Most preclinical studies with ocular cells or dry eye animal models (Section 5.1) have used the finished eye drop products instead of pure trehalose in a control vehicle; no attempts to adjust the concentration has been expressed in these studies either. An unstated consensus thus prevails on the fact that the 3% trehalose concentration, originally determined in one cell culture study [80] and followed by one clinical study with hyperosmolar trehalose eye drops and involving one ethnic patient population with autoimmune diseases [55] (Table 1), is sufficiently established. In another study [43], 1.2% trehalose was found to protect human corneal cells against desiccation-induced cell death, morphological changes, and induction of proinflammatory signaling, whereas 2.4% trehalose caused a significant loss of cell viability within the same time frame. Further, a more recent study showed that 6% trehalose was more efficient than 3% trehalose against UVB irradiation-induced oxidative damage in the rabbit cornea [8], thus approaching the concentrations originally experimented by Matsuo et al. [55,80].

It should be kept in mind that an error of about 10% in the percentage concentration is produced in the absence of information of whether trehalose dihydrate (378 g/mol) or anhydrous trehalose (342 g/mol) is concerned. Currently at least one clinical trial exists in which 2% trehalose dihydrate (ca. 58 mM) was included in combination with other active agents for dry eye [61], and another trial with possibly 1.5% trehalose [62] (as explained in Table 1).

Studies in other medical fields reveal a wider range of usable concentrations of trehalose. A recent review [81] lists several published studies on the effect of trehalose in cellular models of autophagy; the equally preferred concentrations were 50 (1.7%) or 100 mM (3.4%), and even 10–20 mM (0.34%–0.68%) trehalose induced autophagy activators [33]. Another review [82] of Parkinson’s disease models shows that the effective concentrations of trehalose in direct contact with the target cells are in the range 10–100 mM (0.34%–3.4%), in some cases even lower. There seems to be still a limited body of data to conclude that the trehalose concentration in topically administered eye drops should be much higher, although a higher concentration would still be perfectly safe. Eventually, it is the overall composition determining other properties, such as formulation viscosity and retention on the ocular surface, that would bring about the clinical outcome.

### 5.5. Trehalose Patents in Dry Eye

The intention of this chapter is to give a brief overview of patents or patent applications for the use of trehalose in ophthalmologic applications related to dry eye. What aspects are covered by patents and in which countries? Manufacturing patents and other applications of trehalose are beyond the scope of the present review and were excluded. Patent information was collected by using the free and publicly accessible database [69] provided by The European Patent Organisation (EPO).

The patent application WO97/24129 (“Tanaka 1995”; applicant: Rohto Pharmaceutical, Osaka, Japan) appears to be the first to disclose an ophthalmic application for trehalose. The medical treatment “relates to a pharmaceutical composition containing trehalose, which shows protecting effect on cornea and is used safely as an intraocular irrigating solution, eye drops, or eye ointment”. This application was granted in Japan (JP4033510 B2) and lapsed/expired in 2016.

Hayashibara Biochemical Laboratories (Okayama, Japan) was the applicant for an initial European patent EP1192947 (“Matsuo 2000”) claiming an ophthalmic pharmaceutical composition containing trehalose. As the correspondence between the patent office and an applicant can be followed via the Global Dossier feature of the patent database, it appears that the original intention of the applicant to obtain wide protection for treatment of several ophthalmologic conditions was hindered by both the Tanaka 1995 application and a scientific publication of the inventor [80]. These two prior publications thus became obstacles for the novelty of the invention. In the final version EP1192947 (B1) granted in 2006, the patent claims were consequently narrowed to “use of trehalose free of pyrogen in the manufacture of an ophthalmic pharmaceutical composition for the treatment and/or prevention of an ophthalmologic clinical symptom of Sjögren syndrome or an ophthalmologic clinical sign of Sjögren syndrome”. A divisional application EP1649860 for the original Matsuo 2000 patent was submitted to EPO in 2006. Thus, EP1649860 and EP1192947 belong to the same patent family with the same priority date. Again, Tanaka 1995 forced to restrict the divisional application to a somewhat cryptic wording, claiming “use of α,α-trehalose as an effective ingredient for the preparation of an ophthalmic pharmaceutical composition for treating dry eye as an ophthalmologic clinical symptom of Sjögren syndrome”. Both patents are in force in three European countries (Table 2).

Further non-European patents within the Matsuo/Hayashibara patent family share the same priority application with EP1192947 and EP1649860. The claims in each patent granted in various countries are uniform in that the use of trehalose is indicated for treating or preventing symptoms and signs of Sjögren syndrome alone (Table 2). This restriction may not be readily apparent in all cases. In US7732425 (B2) for example, “a method for treating a patient suffering from dry eye” is claimed, by the use of trehalose in an eyewash formulation. Although not explicitly worded in the claims, the patent description itself clarifies that the invention “exerts an outstanding improvement in the ophthalmologic clinical symptoms and signs in Sjögren syndrome, and thus it can be advantageously used in the treatment and/or the prevention of the syndrome.” Obviously, the scope would be restricted to Sjögren syndrome in this case as well. In all cases, the claimed effective trehalose concentration range is from at least 0.01% up to 10% (EP1649860) or to about 30% (US7732425).

It may be concluded that all patents within the Matsuo/Hayashibara patent family protect the use of trehalose for treatment of dry eye related to Sjögren syndrome. These patents share the same priority term, the priority date being Sept. 14, 2000.

Laboratoires Théa and the Medical University of Vienna were granted a European patent EP3110425 in 2019 for an ophthalmic formulation comprising HA and a diholoside, corresponding to the composition of Thealoz^®^ Duo with 0.15% HA and 3% trehalose (a diholoside). The incorporated claims for the molecular weight of HA (100–800 kDa) and viscosity (2–15 mPa s) match those determined for the product by others (see Section 5.3.3). The invention is for use in ophthalmic diseases such as dry eye, and it is based on experimental data showing increased TFT after a single drop application published by Schmidl et al. [66] (Table 1). The priority date of the application is Feb. 28, 2014, and it is in force in most European countries (Table 2).

## 6. Conclusions and an Outlook for Tomorrow

Considering the proceedings reviewed in the preceding chapters, all indicators suggest that trehalose will continue to be a topic of intensive research in ophthalmology and other fields of science, maintaining the exponential trend in the number of medical publications (Figure 1) for the 2020s. These indicators include advancements in manufacturing technologies (Section 2) and consistent effects of trehalose in signaling models for oxidative stress, protein clearance, and inflammation, and in animal models related to dry eye (Section 4 and Section 5.1). To date, trehalose lacks known toxicity to cells and is rather characterized by bioprotection of cells and macromolecules against adverse threats (Section 3, Section 4 and Section 5). The safety and protective effects of trehalose have been a common finding in several clinical trials with topical application in the eyes (Section 5). Active research on the effects of trehalose in neuroprotection and cryopreservation (Section 3) will likely lead to clinical applications in these fields. As trehalose is being considered for the management of neurodegenerative diseases (reviewed in [82]), it is not difficult to imagine that this field is likely to inseminate research in ophthalmic applications as well, such as for treatment of age-related macular degeneration and glaucoma, due to partially shared pathogenetic features [83,84].

Trehalose can be chemically or enzymatically modified to obtain synthetic trehalose analogues to be utilized as nondegradable bioprotectants, therapeutic inhibitors, or bioanalytical probes (reviewed in [85]). For therapeutic activity in a specific application, trehalose or its analogues need to be included in an appropriate formulation and in a sufficient concentration, combined with a suitable delivery option. Development and optimization of these characteristics will facilitate the use of trehalose in a multitude of clinical applications in the field of ophthalmology and beyond.

## Figures and Tables

**Table 1:** Randomized controlled trials with trehalose treatment on ocular surface.

Reference	Clinical Condition (Level of Evidence^1^)	Trehalose and Other Ingredients, Number of Patients	Control Treatment, Number of Patients	Dosing Regimen	Main Findings for Trehalose	Factors of Possible Influence
**Dry Eye**						
Matsuo et al. 2002 [55]	Moderate to severe dry eye (level 2)	100 mM (3.4%),^2^ n = 18 200 mM (6.8%), n = 16	Saline, n = 18 Saline, n = 16	6 times/day for 4 weeks in randomized eyes	Increased TBUT (100 mM only), decreased staining scores. No improvement in dry eye symptom score. No adverse effects.	Eye drop osmolality 1.3 and 1.8 times higher than control (hyperosmolar) Autoimmune patients.
Matsuo et al. 2004 [56]	Moderate to severe dry eye (level 2)	100 mM (3.4%),^2^ n = 33 200 mM (6.8%), n = 3 (continuation from Matsuo et al. 2002 trial [55])	0.1% HA (Hyalein), weeks 1–4, n = 18 Hydroxyethyl cellulose (Mytear), weeks 5–8, n = 18	4 times/day for 4 weeks in both eyes in randomized cross-over design	Increased TBUT, decreased staining scores. No improvement in dry eye symptom score at 4 weeks but improved at 8 weeks. No adverse effects.	Eye drop osmolality 1.3 and 1.8 times higher than control (hyperosmolar) Mostly Sjögren syndrome patients.
Pinto-Bonilla et al. 2015 [57]	Moderate to severe dry eye (level 1)	3% trehalose + 0.15% HA (Thealoz^®^ Duo), n = 17	Hydroxypropyl guar + polyethylene glycol + propylene glycol, preservative (Systane^®^), n = 17	5 times/day for 7 days in both eyes in randomized cross-over design	Improved global satisfaction, dry eye symptoms and their impact at work. Improved OSDI and staining scores with no significant difference to control. No adverse effects.	Open-label (no masking). Short duration.
Chiambaretta et al. 2017 [58]	Moderate to severe dry eye (level 1)	3% trehalose + 0.15% HA (Thealoz^®^ Duo), n = 52 (46 per protocol)	0.18% HA (Vismed^®^), n = 53 (45 per protocol)	3–6 times/day for 84 days in both eyes	Improved Oxford grading score at 35 days (per-protocol dataset). Improved subjective (patient, investigator) evaluation scores. Improved TBUT, Schirmer, van Bijsterveld, and conjunctival hyperaemia score with no significant difference to control. No difference in global tolerability.	No masking of patients.
Doan et al. 2018 [59]	Moderate to severe dry eye (level 1)	3% trehalose + 0.15% HA (Thealoz^®^ Duo), n = 52	0.18% HA (Vismed^®^), n = 53	3–6 times/day for 84 days in both eyes	Improved OSDI at 84 days.	A post hoc analysis of Chiambaretta et al. 2017 [58] No masking of patients.
Fondi et al. 2018 [60]	Moderate to severe dry eye (level 1)	3% trehalose + 0.15% HA + 0.25% carbomer + sorbitol (Thealoz^®^ Duo Gel), n = 45	3% trehalose + 0.15% HA (Thealoz^®^ Duo) and 3% trehalose + 0.15% HA + 0.25% carbomer + sorbitol (Thealoz^®^ Duo Gel), n = 45	Daytime: as frequently as needed Night-time: Thealoz^®^ Duo Gel	Improved TBUT, staining scores and quality of sleep with no significant difference between groups. No improvement in Schirmer I. Reduced frequency of instillations for Thealoz^®^ Duo Gel. No adverse effects.	Screening data incompletely reported. No masking of patients. Short duration.
Panigrahi et al. 2019 [43]	Moderate or severe dry eye symptoms and mild signs (level 2)	3% trehalose + 0.1% HA, preservative (Trehalube^TM^), n = 9	0.5% CMC, preservative (Lubrex^®^), n = 9	2 times/day for 30 days in randomized contralateral eyes	Improved OSDI with no significant difference between groups. Improved TBUT. No improvement in Schirmer 1 or 2. Reduced tear fluid MMP-9, MMP-2, MCP-1, and MMP-9/TIMP-1 ratio.	No masking of investigators. OSDI measured separately for contralateral eyes.
Laihia et al. 2019 [61]	Moderate or severe dry eye (level 1)	2% trehalose + 0.1% sacha inchi seed oil + 0.2% HA + glycerol, n = 26	0.2% HA, n = 26	3 times/day for 30 days	Improved ocular protection index (TBUT/interblink interval). Improved TBUT, staining and redness scores, hyperosmolarity and OSDI with no significant difference to control. No adverse device effects.	Other active agents used with trehalose.
Downie et al. 2019 [62]	Mild to severe dry eye (level 1)	1.5% trehalose^3^ + flaxseed oil + CMC + glycerol + castor oil + levocarnitine + erythritol (Refresh Optive^®^ MEGA-3), n = 120	CMC + glycerol + castor oil + L-carnitine + erythritol (Refresh Optive^®^ Advanced), n = 122	≥ 2 times/day for 90 days	Improved combined ocular staining score (days 7, 30, 60, and 90), corneal staining (day 90) and conjunctival staining (day 30). Improved burning/stinging (day 60). Improved OSDI, TBUT and staining scores with no significant difference to control. Conjunctival hyperemia (1.7%), instillation site pruritus (1.7%).	Patients with OSDI score > 65 were excluded. Effects of trehalose and flaxseed oil cannot be distinguished.
**Ocular Surgery**						
Mateo Orobia et al. 2017 [63]	LASIK post-treatment (level 1)	3% trehalose (Thealoz^®^) and 0.15% HA (Hyabak^®^), n = 7	0.15% HA (Hyabak^®^), n = 6	Thealoz^®^ 4 times/day + Hyabak^®^ 5 min later in both eyes Hyabak control: every 2 h for 10 days, 6 times/day for 3 months in both eyes	Improved SANDE severity and frequency scores. Improved staining scores at 30 and 90 days. No improvement in osmolarity, TBUT, visual acuity, and OSDI.	Small number of patients with 12/13 males. No masking of patients.
Caretti et al. 2019 [64]	Cataract surgery and mild to severe dry eye (level 1)	3% trehalose + 0.15% HA + 0.25% carbomer + sorbitol (Thealoz^®^ Duo Gel), n = 30	0.15% HA (Hyabak^®^), n = 30	2 times/day for 1 month in operated eye	Improved TBUT, OSDI and patients’ global satisfaction score. Improved staining scores and visual acuity with no significant difference to control. No improvement in Schirmer.	
Vagge et al. 2019 [65]	Strabismus surgery (level 2)	Chloramphenicol/betamethasone and 3% trehalose + 0.15% HA + 0.25% carbomer + sorbitol (Thealoz^®^ Duo Gel), n = 31	Chloramphenicol/betamethasone	3 times/day for 4 weeks	Improved blurred vision at 1 and 4 weeks, other symptoms at 1 week. Improved conjunctival redness with no significant difference to control.	Open-label (no masking). Numerical rating of symptoms assessed separately for contralateral eyes.
**Tear Film Dynamics**						
Schmidl et al. 2015 [66]	Mild or moderate dry eye (level 1)	3% trehalose + 0.15% HA (Thealoz^®^ Duo), n = 20	0.15% HA (Hyabak^®^), n = 20 Saline (Hydrabak^®^), n = 20	1 drop in one eye	Increased TFT at 10–240 min. No improvement in TBUT and Schirmer I at 240 min. No adverse effects.	Custom-built OCT system. One dose only.
Wozniak et al. 2017 [67]	Moderate or severe dry eye (level 1)	3% trehalose + 0.15% HA + 0.25% carbomer + sorbitol (Thealoz^®^ Duo Gel) n = 20	0.4% polyethylene glycol + 0.3% propylene glycol + hydroxypropyl guar + sorbitol (Systane^®^ Gel), n = 20 0.2% HA + sorbitol (Hylo^®^-Gel), n = 20	1 drop in both eyes	TFT increase (+66%) lower than with Systane^®^ Gel (+156%) but higher than with Hylo^®^-Gel (+33%) control at 10-min peak. Increased TFT (less than +20%) at 60 and 120 min. Increased TBUT in all groups at 360 min No increase in Schirmer I at 360 min.	Same OCT system as in Schmidl et al. 2015 [66] but with higher baseline values. One dose only.
Karaca et al. 2019 [68]	Mild or moderate dry eye (level 2)	3% trehalose + 0.15% HA + 0.25% carbomer + sorbitol (Thealoz^®^ Duo Gel), n = 56	0.3% HA (Vismed^®^ Gel), n = 66	1 drop in one eye	Lower TMH and TMD for 10–120 min. Lower patient satisfaction at 240 min. No improvement in TBUT, tear osmolarity and Schirmer I at 240 min.	All patients were with primary Sjögren syndrome. One dose only.

^1^ Ref. [54]; ^2^ Percentage calculated from molar concentration of anhydrous trehalose; ^3^ Concentration not disclosed in publication; derived from US patent 10,279,005 B2 [69].

**Table 2:** Summary of granted patents on trehalose for dry eye.

Patent	General Field of Protection ^1^	Designated Country
JP4033510 (B2)	Trehalose for protecting cornea and used as eye drops	JP
EP1192947 (B1)	Trehalose in treatment and/or prevention of symptom or sign of Sjögren syndrome	DE FR GB
EP1649860 (B1)	Trehalose in treatment of dry eye as a clinical symptom of Sjögren syndrome	DE FR GB
US6555526 (B2)	Ophthalmic pharmaceutical composition with trehalose for treatment and/or prevention of symptom and sign in dry eye in Sjögren syndrome	US
US7732425 (B2)	Method for treating dry eye (Sjögren syndrome) with trehalose in the form of eyewash solution	US
CA2355814 (C)	Ophthalmic pharmaceutical composition with trehalose for treatment and/or prevention of symptom or sign of Sjögren syndrome	CA
AU781975 (2001065453) (B2)	Ophthalmic pharmaceutical composition with trehalose for treatment or prevention of symptom or sign of Sjögren syndrome	AU
KR100776124 (B1)	Trehalose in treatment and/or prevention of symptom and sign in dry eye in Sjögren syndrome	KR
TWI291350 (B)	Trehalose in treatment or prevention of symptom and sign of Sjögren syndrome	TW
JP4982643 (B2)	Trehalose in treatment and/or prevention of symptom or sign of Sjögren syndrome	JP
EP3110425 (B1)	Trehalose (as a diholoside) with 100–800 kDa HA for treating ophthalmic diseases such as dry eye	Most EPO countries

^1^ Descriptive presentation of patent claim(s).

## Abbreviations

AMPK	monophosphate-activated protein kinase
AP-1	activating protein-1
ARE	antioxidant response element
CB	combination preparation containing chloramphenicol and betamethasone
CMC	carboxymethylcellulose
EPO	European Patent Organisation
HA	hyaluronic acid
hCEC	human corneal epithelial cells
HSP	heat shock protein
KEAP1	kelch-like ECH-associated protein 1
LASEK	laser subepithelial keratomileusis
LASIK	laser-assisted in situ keratomileusis
MeSH	Medical Subject Headings
MMP	matrix metalloproteinase
mTOR	mechanistic target of rapamycin
NEI	National Eye Institute/Industry grading scale
NFE2L2	nuclear factor erythroid 2-related factor 2
NF-κB	nuclear factor-κB
OCT	optical coherence tomography
OSD	ocular surface disease
OSDI	Ocular Surface Disease Index
PBS	phosphate-buffered saline
ROS	reactive oxygen species
SANDE	Symptom Assessment in Dry Eye
SLC2A8	Solute Carrier Family 2 Member 8
TBUT	tear film break-up time
TFT	tear film thickness
THC	combination preparation containing trehalose: hyaluronic acid: carbomer and sorbitol
TMD	tear meniscus depth
TMH	tear meniscus height
TNF-α	tumor necrosis factor-α
Tret1	trehalose transporter-1
UPS	ubiquitin proteasome system
UVB	ultraviolet-B
